# Player Experience Evaluation in Game-Based Systems for Older Adults

**DOI:** 10.3390/s24186121

**Published:** 2024-09-22

**Authors:** Johnny Alexander Salazar-Cardona, Bryjeth Ceballos-Cardona, Patricia Paderewski-Rodriguez, Francisco Gutiérrez-Vela, Jeferson Arango-López

**Affiliations:** 1 Departamento de Sistemas e Informática, Facultad de Ingenierías, Universidad de Caldas, Calle 65 # 26-10, Edificio del Parque, Manizales 170004, Colombia; jeferson.arango@ucaldas.edu.co; 2 Departamento de Lenguajes y Sistemas Informáticas, ETSI Informática, Universidad de Granada, 18071 Granada, Spain; ybceballosc@correo.ugr.es (B.C.-C.); patricia@ugr.es (P.P.-R.); fgutierr@ugr.es (F.G.-V.)

**Keywords:** older adults, game-based system, player experience, playability, HCI

## Abstract

Significant efforts are currently being made to improve the quality of life of the older adult population. These efforts focus on aspects such as health, social interaction, and mental health. One of the approaches that has shown positive results in several studies is the application of game-based systems. These systems are not only used for entertainment, but also as tools for learning and promoting positive feelings. They are a means to overcome loneliness and isolation, as well as to improve health and provide support in daily life. However, it is important to note that, while these experiences are gradually being introduced to the older adult population, they are often designed with a younger audience in mind who are assumed to be more technologically proficient. This supposition can make older adults initially feel intimidated when interacting with this type of technology, which limits their ability to fully utilize and enjoy these technological solutions. Therefore, the purpose of this article is to apply a game experience and fun evaluation process oriented toward the older adult population based on the playability theory of human–computer interaction in virtual reality game experiences. This is expected to offer highly rewarding and pleasurable experiences, which will improve engagement with the older population and promote active and healthy aging.

## 1. Introduction

Older adults have different motivations than younger people, because people change their social orientations and goals over the course of their lifetime [1]. Older adults perceive future time as limited, so they prioritize emotional goals and avoid experiences with aversive effects, such as time pressure to complete challenges or the frustration of not achieving some challenges due to their high complexity. In contrast, young people perceive time as unlimited, so they prioritize learning new things and do not care about aversive experiences with duration limitations and a high difficulty. The latter, on the contrary, drive them and motivate them to play [1,2].

The older population is a heterogeneous group [3], but it shares some behavioral patterns that allow us to characterize its motivations [4]. Older adults want to stay active, learn new things, and contribute to society. These motivations can be intrinsic or extrinsic. Intrinsic motivations are those that arise from within the person, such as the pleasure of learning or the desire to help others. Extrinsic motivations are those that come from the outside, such as financial reward or social recognition [5].

Research on older adults’ motivations for playing games has focused on four main areas: “Encouraging physical activity and rehabilitation processes”, with serious games being used to help older adults stay active and improve their physical health [6,7,8,9]; the “Identification of motivations in older adults in traditional digital games”, such as computer games, console games, and mobile games [10,11,12,13,14,15]; the “older adults” motivations in the use of technology”, not focusing on the game experience but on their technological peripherals [16]; finally, the “Application of models”, where some studies have applied existing models, such as the self determination theory (SDT) model [17,18,19,20] and the emotional processes and self-regulation model [21] to identify older adults’ motivations in serious games.

Technology acceptance in older adults has opened a space that focuses on understanding how this population adopts and uses new technologies. Models such as the technology acceptance model (TAM) [22] and the unified theory of acceptance and use of technology (UTAUT) [23] have been widely applied to assess these aspects. According to the TAM, perceived usefulness and perceived ease of use are key determinants of technology adoption. On the other hand, the UTAUT includes significant additional factors, such as social influence and facilitating conditions.

For older adults, these factors are essential because technologies must be perceived as beneficial and easy to use to be accepted. In addition, the state of the technology infrastructure, continuous training, and the opinions of people close to them influence the perception of how technology can be effectively and easily integrated into their daily practices.

In previous research results, a game-based systems (GBS) evaluation process has been defined that offers design recommendations and means to identify potential design problems, to assess playability and fun, and, finally, to evaluate the experience perceived by older adults [24]. Regarding this process, it has been applied in the design of pervasive game experiences with tangible interaction, obtaining satisfactory results and identifying many elements that can be improved in initial designs and prototypes [25]. The process for the selection and evaluation of a virtual-reality game experience that could be used by the older adult population was also applied. Finally, this entire evaluation process is published on the PL/PX web platform hosted at https://plpx.johnnysalazar.net/ [26].

In this work, the phase of evaluating the experience perceived by the older adult in the previously established evaluation process has been applied and validated. For this, a previously chosen game experience was used and evaluated by an older adult population through a series of phases. To detail the process, this document is organized as follows: Section 2 contextualizes the defined evaluation process and its previous application of stages 1 and 2 of the process, where the game was selected and evaluated by experts; Section 3 describes the detailed application of stage 3 of the evaluation process, oriented toward the perceived experience based on the established methodological process; finally, in Section 4, a discussion of the results is carried out, the conclusions of the study are presented, and possible lines of future work are raised.

## 2. Background

Usability refers to the ability of a product to be used effectively, efficiently, and satisfactorily by users in a specific context [27]. Beyond effectiveness, efficiency, and satisfaction, user experience (UX) refers to how people perceive and respond when interacting with a product or service [28]. This can generate positive or negative responses before, during, or after use. In the field of GBS, usability evaluations are not always completely objective due to subjective elements inherent to the game experience [29,30]. The concept of playability then emerged, which focuses on analyzing, in-depth, the quality of the GBS, providing a more accurate measure of the fun it offers [31,32].

It is established that only considering usability is not enough to evaluate player satisfaction, especially in video games, since their main objective is entertainment [33]. Fun is subjective and related to the individual characteristics of each player, which complicates assessment [30]. Game experiences involves cognitive, emotional, and social components that emerge in the interaction between players and the game experience [34]. From this arises the notion of player experience (PX), which focuses on the emotions, satisfaction, and engagement of the individual when interacting with a GBS [35]. In this context, it can be concluded that playability refers to improving the design of the game itself, while PX is oriented towards improving the quality of the player’s experience and feelings [30,36].

Currently, there are different UX assessment instruments that are applied to the older adult population to measure their experiences, opinions, and acceptance of GBS. These instruments, although valid, present limitations in GBS as explained above and have not been fully adaptive to the needs and particularities of this population. The lack of evaluations adjusted to the particularities of older adults may be due, in part, to the lack of a detailed characterization of tastes, motivations, and preferences that would allow the generation of evaluation instruments adapted to the needs and particularities of this population.

Some cases of existing applications used to measure the quality of GBS as a product in older adults have made use of traditional usability tools such as the “System Usability Scale” (SUS) [37]. Some examples of this are the application of exergames where older adults had to play a game and answer a questionnaire to obtain information on effectiveness, efficiency, and satisfaction [38,39,40,41,42,43,44,45,46,47]. There are also cases of SUS application in games oriented toward cognitive training [48].

Another commonly used evaluation tool for evaluating the UX in game-based systems has been the “Technology Acceptance Model” (TAM) [49], used for feedback on the positive effect, durability, aesthetics, and sensory appeal of game experiences. This, like the SUS, is not oriented toward this type of system, but some examples of the application of this model in older adults were carried out with the game “Balloons Rescuer” [50], shooting games [51], and games for cognitive training [43]. The application of the “Unified Theory of Acceptance and Use of Technology” is also highlighted (UTAUT2) [52] to measure acceptance of the technological use of a game experience [53,54]. Finally, an adjusted UX or similar evaluations are found in older adults for game-based systems through questionnaires or by analyzing their gestures during the experience [50,55,56].

There are also more accurate assessments focused on the older adult population, such as PX evaluation [36] of their interaction with game-based systems [57,58,59,60,61]. In these evaluations, the use of evaluation instruments such as the “Game Experience Questionnaire” (GEQ) [62], the “Player Experience of Need Satisfaction” (PENS) [63], “Intrinsic Motivation Inventory” (IMI) [64], and “Positive and Negative Affect Schedule” (PANAS) is evident [65]. Although these may be valid, they also do not adjust to the needs and particularities of the older adult population [30].

### 2.1. Influencing Factors in the Measurement of Technological Acceptance in Older Adults

Technology acceptance has become a field of research that seeks to understand the determining factors in the user’s intention to use a technology. However, these factors cannot be fully determined without considering a few specific characteristics of the population where the technology will be implemented. Technology acceptance models such as the TAM, by themselves, are inadequate to predict and explain technology acceptance in older adults [66]. However, it allow for the inclusion of additional factors that depend on the target technology, the potential users, and the context.

**Figure 1:**
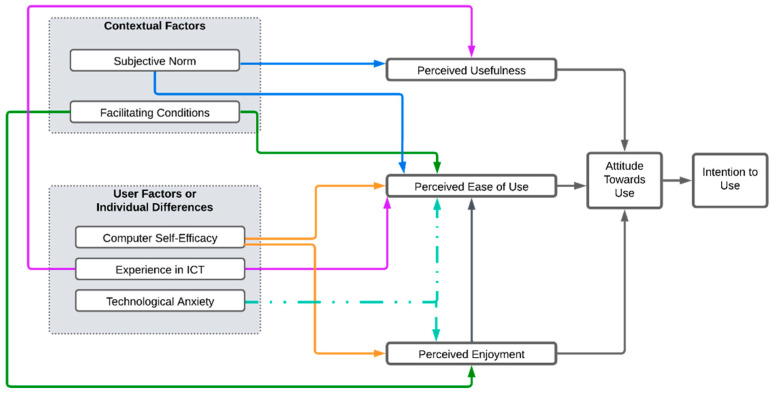
Influencing factors in technological acceptance in older adults.

In this sense, it is proposed to adapt the TAM by maintaining the perceived usefulness and perceived ease of use as dependent variables that predict the attitude towards use, which in turn predicts the intention to use the technology. In addition, it is proposed to incorporate the perceived enjoyment variable into the model, which acts as a predictor of the perceived ease of use and attitude toward use. It is also important to consider external factors that affect the independent variables of the model, such as contextual factors that determine variables such as the subjective norm and facilitating conditions; and user factors or individual differences that affect variables such as computational self-efficacy, experience in handling technology, and technological anxiety (See Figure 1).

Several studies [67,68,69] have shown that factors associated with the context in which the user lives have a significant influence on technology acceptance. The subjective standard, defined as the perceived social pressure to perform or not to perform a behavior [70], is related to the influence that reference groups exert on the performance of a specific behavior. In this sense, social influence significantly affects older people’s intention to use technology, and the social support of family members, such as children and grandchildren, has a notable effect on their perception of usefulness and ease of use.

Regarding facilitating conditions, these do not directly influence the attitude towards technology use, but do so through perceived ease of use [71], as these conditions include technological infrastructure and available technical support which facilitate both access to and use of the technology.

User factors or individual differences impact how older adults feel prepared to use technology. Computational self-efficacy is defined as a user’s judgment of their ability to use a technology, prior to having hands-on experience with the new technology. This judgment does not relate to the skills the user has, but to their perception of what they can do with their skills [72]. In older adults, it is the judgment they have about how they use and manipulate technology that impacts their perceived ease of use and perceived enjoyment; people with high self-efficacy are not deterred by obstacles and take on challenges that enable them to use technology effectively.

Technology experience refers to the user’s prior experience with a technology; high experience is associated with a higher perceived ease of use, allowing more experienced older adults to better see the usefulness of the technology tool.

Technology anxiety is defined as the set of complex emotional reactions that are activated in individuals when interpreting technology as a threat. It is a person’s tendency to experience unease about the impending use of a technology [73]. Unlike the other variables, it acts as a barrier for the older population; anxiety increases with age and manifests as a personality trait that leads to perceiving technology as a threat. This variable has a negative influence on the perceived ease of use and perceived enjoyment, affecting the attitude towards use and, consequently, the intention of older adults to use a given technology.

In addition, the model shows how perceived enjoyment positively influences the perceived ease of use, as it measures the degree to which the activity of using a technology is perceived as a personal pleasure. This can help reduce older adults’ anxiety and make them feel more confident in their ability to successfully execute the required actions.

### 2.2. Evaluation Process Model

The proposed playability and PX evaluation process is carried out using heuristics, which are used to identify potential usability issues based on a previously established set of heuristics [74]. Heuristics are a set of rules that guide how to perform such evaluations, and can be used to identify problems, and evaluate playability and player experience, which is how it is applied in the field of usability and user experience UX. These general heuristics can be complemented with checklists to facilitate and refine the review and evaluation of a game in a more rigorous way.

**Figure 2:**
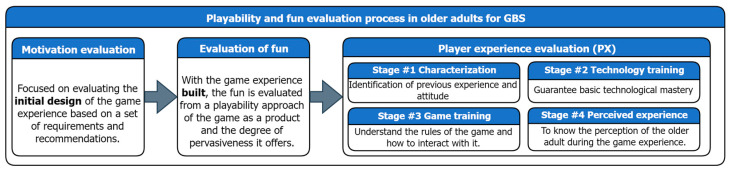
Summary of the evaluation process defined in [24].

In the case of older adults, the process should consider the evaluation of the game experience from an approach focused on motivational elements involved in the initial design and evaluation of the fun from a playability approach in regard to the game experience built. Finally, the PX should be evaluated by an older adult when interacting with the game experience [24]. Based on the above, the evaluation process of game experiences and fun in older adults has been defined, which considers all the elements previously mentioned (see Figure 2). It should be noted that this document will only focus on validating the application of stage 3, called “player experience evaluation”. The validation of stage 1 and 2 can be found in [24].

### 2.3. Preliminary Stages: Motivation and Fun Evaluation

To validate the proposed evaluation process, a PL/PX web platform was developed to provide a tangible means of applying the entire evaluation process, offering evaluation formats and tools in each of the stages, with the possibility of obtaining metrics and indicators in real time. For the validation of the first stage (motivation evaluation), a design and initial prototype of a game experience called “The memories chest” was developed in collaboration with the “Pontificia Universidad Católica de Valparaíso” which offered pervasiveness and tangible interaction to be friendly with the older adult population. This was evaluated by a total of eight experts who performed a heuristic evaluation using our proposal, identifying a total of 58 potential problems and possibilities for improvement [25].

Subsequently, to execute the second stage of the process, the “evaluation of the fun of the game experience”, it was essential to have a prototype game-based system that was fully mature and functional. Although initially it was considered to use the designed game experience “the memories chest” to validate this stage, it was finally discarded. The reason behind this decision is that, for the next stage of “evaluation of perceived experience”, a more mature game experience is required, which allows obtaining as much information as possible from the older adults.

Therefore, in the “fun evaluation” stage, it was decided to select different games that could be adequately adjusted and adapted to the particularities of the older adult population. The objective is to ensure that the game experience is as enriching and fun as possible for this specific group, considering their needs and preferences. Thus, choosing games that are more suitable for this purpose will allow more accurate and valuable results to be obtained during the evaluation of the experience perceived by the older adults involved. This change ensures that the evaluation process is robust and provides relevant information for the development of highly rewarding game experiences for this segment of the population. In the process, the game “Beat Saber” was selected and evaluated by eight experts using our tool, obtaining favorable playability results. The results of the experts’ evaluation can be found in [24].

### 2.4. Synthesis

**Figure 3:**
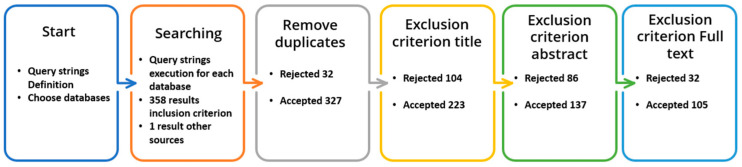
Systematic literature review process.

The process of game evaluation and creating fun experiences for older adults using GBS has had an extensive process of definition, evidenced in our previously conducted research. Initially, a detailed and exhaustive systematic literature review was carried out [75], in which detailed information on the different motivational elements in game-based systems for older adults was obtained. This process aimed to answer different research questions addressed to older adults, among which was their acceptance of the use of games through technology, and which game mechanics and dynamics were the most used and accepted by this population. To carry out this research, the methodology established by Kitchenham and Charters was applied [76], which defines a series of steps or phases for the application of systematic reviews in the field of software. Following the base methodology, a series of inclusion and exclusion criteria were defined to reduce the total number of articles, with the reduction process being carried out as shown in Figure 3.

**Figure 4:**
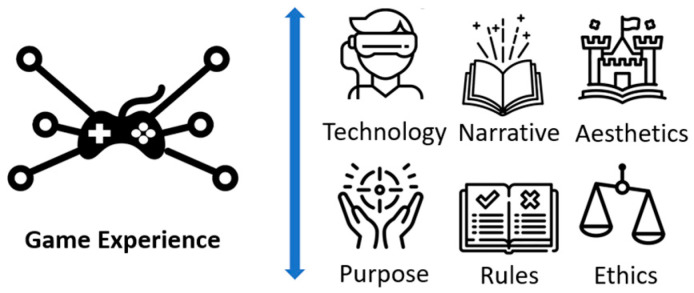
Transversal game elements to offer better PX [24].

**Figure 5:**
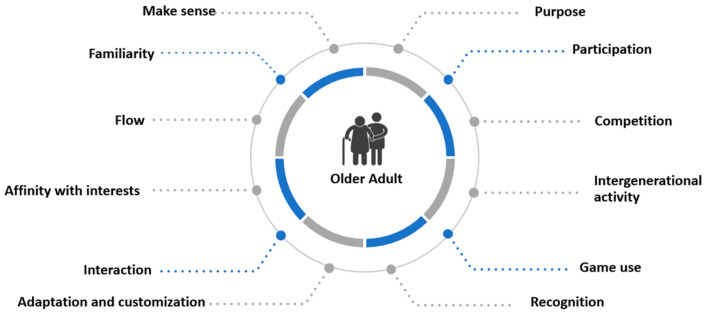
Characterization of motivational aspects in older adults [24].

Based on the findings of this systematic review, we applied the methodology defined by Quiñonez et al. [77], in which it is established in detail how to perform a formal process of definition of heuristics with their respective validations. All the findings of the review were treated in accordance with the methodology, resulting in different aspects of the game elements [78,79] (see Figure 4) and motivation aspects [4] (see Figure 5) that help to generate engagement with older adults, being represented in different heuristics and checklists that detail the elements that should be incorporated in the design of a GBS. Then, based on the different heuristics identified, the evaluation process for game and fun experiences was established, composed of three main stages: motivation evaluation, evaluation of fun, and player experience evaluation.

#### 2.4.1. Motivation Evaluation

Regardless of the type or nature of the GBS, when designing a gaming experience for older adults, it is important to conduct an initial review to ensure that it is aligned with the motivations of this demographic. This review should be conducted by a set of experts or individuals with experience in conducting heuristic evaluations, ideally also with expertise in GBS. To achieve this, use should be made of the previously established heuristics and checklists focused on motivation and game elements, which are available on the PL/PX web platform [26].

**Figure 6:**
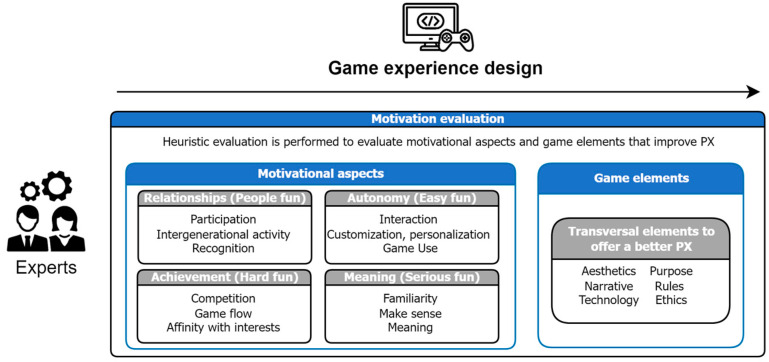
Motivation evaluation in the design of the GBS [24].

The heuristics presented and the related checklist can be considered not only as guidelines in the heuristic evaluation process, but also as design recommendations that will allow a better acceptance of the GBS by older adults. In addition, it is clarified that the heuristic evaluation at this stage is not to generate metrics and indicators, nor to evaluate the quality of the product; it is a means to identify potential problems and opportunities for improvement in the design of the GBS (see Figure 6).

#### 2.4.2. Evaluation of Fun

After performing the evaluation of the motivations in the design of the game experience, an evaluation of the fun offered by this already-built experience must be carried out, focusing on the playability property. Currently, there are several instruments to evaluate the playability of a game-based system. Among these instruments, one of the most complete to date is the tool based on the playability facets and attributes established by Gonzales and Gutiérrez [29].

**Figure 7:**
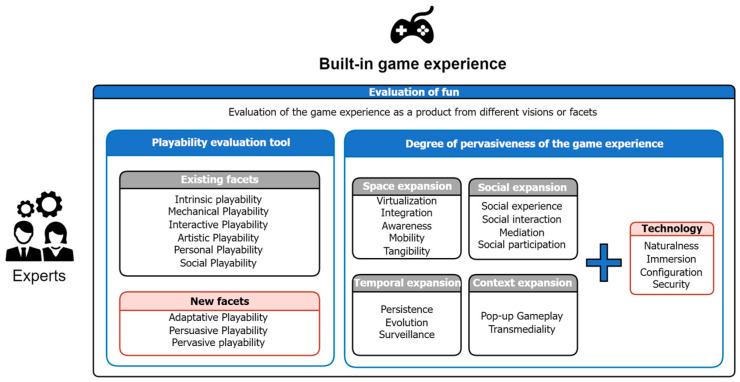
Evaluation of fun in the gaming experience built [24].

An extension of this evaluation instrument has been made to adapt it to the older adult population, considering our design recommendations related to motivations and identified game elements. Unlike the previous stage, where a heuristic evaluation focused on the motivational aspects of the initial game design, in this case, a heuristic evaluation of the completed game or a functional prototype is carried out. The objective is to evaluate the quality of the game as a product and thus determine how much fun it is, by obtaining metrics and indicators, which will provide an objective, measurable result adapted to the specific needs and characteristics of the older adult population (Figure 7).

The new evaluation instrument established has some major differences with respect to the original instrument, these being the following:

- Although the six facets of playability established in the original model are used, three new facets have been defined, namely adaptive playability, pervasive playability, and persuasive playability;
- All the checklists for each previously existing facet were completely redesigned to adjust to the characteristics and particularities of older adults, from the perspective of motivations and the particularities of the game experience;
- The new facet of pervasiveness not only allows us to analyze the degree of pervasiveness of the game experience with respect to playability attributes, but also offers a new analysis focused on the different extensions and properties of pervasiveness;
- The pervasiveness analysis has been extended to include a more detailed exploration of its facets, enabling a technological approach that encompasses fundamental properties such as naturalness of interaction, immersion, configuration, and security;
- It should be noted that, within the original evaluation instrument, mechanical playability was the part that underwent the fewest changes. This is because this evaluation covers response times and fluidity, aspects directly related to the technological implementation, which remain the same regardless of the target population.

#### 2.4.3. Player Experience Evaluation

Finally, once the evaluation of the fun of the game experience has been carried out from a playability approach, the next step is to generate an evaluation of the player’s experience. However, because the older adult population is not familiar with technology and game-based systems, it is necessary to approach this process with special care. To ensure that older adults can interact with the game experience autonomously, both technological training and experience of the game rules must be prioritized.

To avoid psychological overload for the older adult, it is essential to use an assessment instrument that is not extensive or complex, or that assesses multiple factors simultaneously, as this could confuse the older adult. In addition, it is important to use appropriate terminology so that the older adult clearly understands the questions and can provide objective answers. For this reason, a compact model focused on the perceptions from the player’s perspective was developed, based on the playability evaluation instrument.

Although this evaluation is concise, it addresses the nine facets of playability and all the associated transversal attributes. In this way, a complete vision is achieved and allows contrasting the results of the older adult with those obtained in the evaluation of the experts from the point of view of the game experience as a product, that is, the evaluation of the fun it can offer. In the following section, the “player experience evaluation” stage, which is the focus of this paper, will be discussed in more detail.

## 3. Methodology

**Figure 8:**
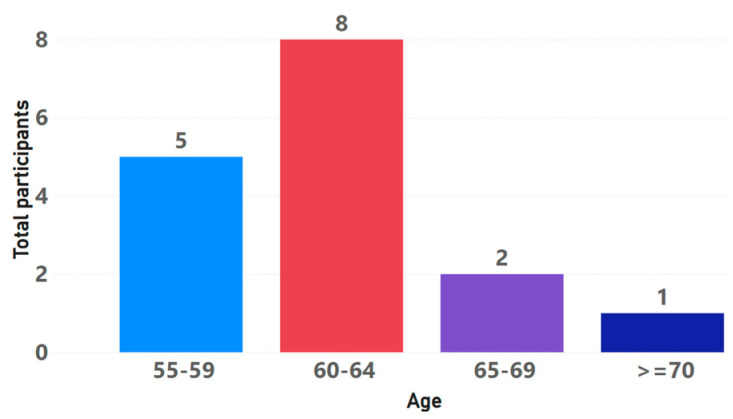
Age range of participants.

For the player experience evaluation, with the game chosen and evaluated by the experts, we then proceeded to execute the last stage called “player experience evaluation”. For this, a total of 16 participants between 55 and 75 years old were recruited (see Figure 8). The participants included seven men and nine women of all educational levels. All participants had full cognitive capacity and mobility, without any physical limitation that prevented them from moving around autonomously. In addition, none of them had previously had experience with virtual reality glasses. Only two of the participants had experience with digital games, as they used to play occasionally with their children and grandchildren.

It is important to explain that, although 16 participants were recruited and their age distribution is shown, only 15 older adults participated in the process, because one of the participants, specifically the older adult who was over 70 years old, stated that in her daily life she felt overwhelmed by images, such as those of movies or similar, and large screens, so it was decided not to carry out the validation process with this participant. For this reason, only 15 participants were considered in the subsequent analysis and results.

In this last stage, oriented toward the evaluation of the player experience, with the aim of ensuring that the older adults can fully understand and enjoy the game experience offered without this being a psychological burden, a process divided into four phases has been established in which a total of 57 questions are asked, which seek to evaluate not only the different facets of playability, but also to obtain information about the adoption, acceptance, and training of the game experience and the technology used. In addition, these questions provide information on the fun, intention of future use, and usefulness of the experience.

**Figure 9:**
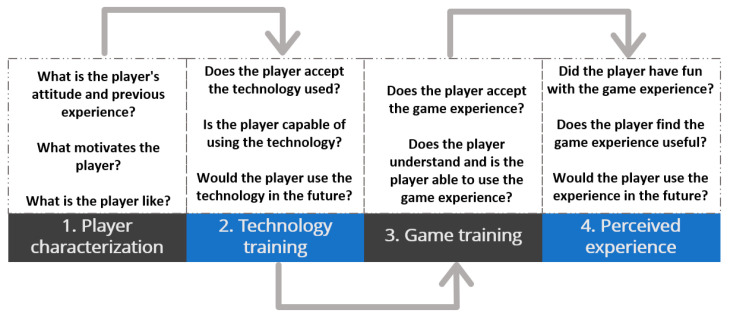
Phases of the player experience evaluation [24].

These stages seek to ensure a comfortable and gradual interaction with the GBS, as well as constant feedback from the user. The four phases of the process are the following: characterization, technological training, game experience training, and evaluation of the perceived experience (see Figure 9). It should be noted that the evaluation instruments that can be used in each of these phases are available on the PL/PX web platform [26].

**Figure 10:**
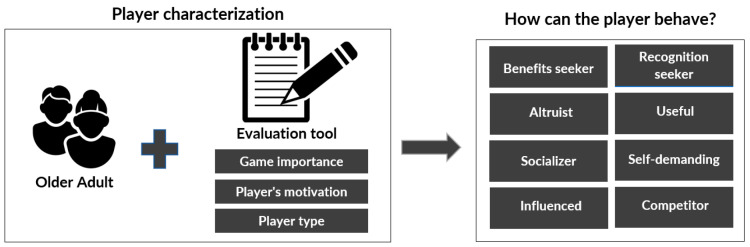
Player characterization phase [24].

In the first phase, called “player characterization”, it is essential to know the player who will interact with the GBS and to carry out a transversal evaluation of the player’s experience. To achieve this, prior to any interaction with the GBS technology and its mechanics, it is necessary to perform an initial characterization. It is important to highlight that this characterization does not aim to create specific metrics or indicators for the older adult, but to understand their previous experience and attitudes towards the GBS at the individual and social level, as well as their motivations and also to try to identify the type or profile of the player [80] (see Figure 10).

For the second phase, called “technology training”, given that the game experiences being addressed are technological and have a certain degree of pervasiveness, it is recommended to provide initial technology training for the participants to understand the basics needed to manipulate the game peripherals. Older adults are faced with devices such as VR glasses or voice assistants such as Alexa or Google Assistant, which may increase the difficulty in their interaction with the GBS if they have no prior experience with these technologies.

**Figure 11:**
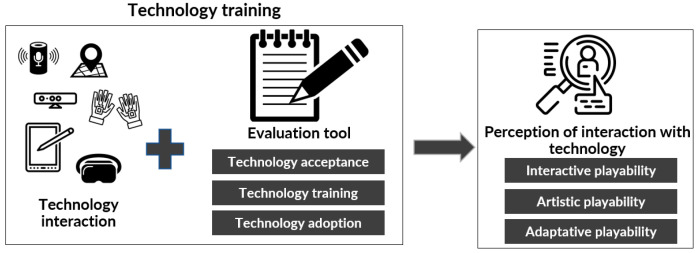
Technology training phase [24].

To achieve this training effectively, it is essential to start with a serene and friendly presentation of the technological device to be used. Its purpose, how to activate it, and how to interact with it in an environment that differs from the usual game experience should be clearly explained. Assistance should be calm and relaxed to facilitate the learning process. For example, if a device such as Alexa is to be used, the older adult must be clear that they must first say “Alexa” or the name configured for activation, otherwise the game experience may be difficult and will frustrate the older adult (see Figure 11).

After receiving training in technology and achieving a basic mastery in this area, the third phase, called “Game training”, is executed, focused on instructing the older adult in the game so that the player understands its rules and the interaction process. It is essential to provide the player with context about the history of the game and the characters involved. Given that there has already been previous interaction with the peripherals, this training process should be simpler, allowing the player to focus fully on the dynamics and mechanics of the game.

**Figure 12:**
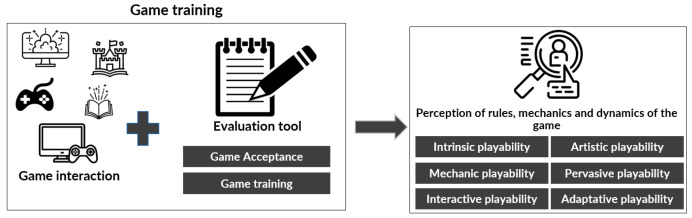
Game training phase [24].

To achieve effective training, it is essential to provide constant accompaniment to the older adult, in a slow and calm way, starting from an easy level and, if necessary, gradually increasing the difficulty according to the skills and progress of the older adult. The success of this phase is reached when the older adult achieves a state of autonomy and independence in interacting with the GBS. At this point, the player should be able to start, pause, or resume the game experience independently, or at least interact with the game and achieve victory. To achieve this, GBS features such as the game difficulty or facilitators may need to be adjusted, provided that the older adult is able to interact with the game and achieve victory appropriately (see Figure 12).

**Figure 13:**
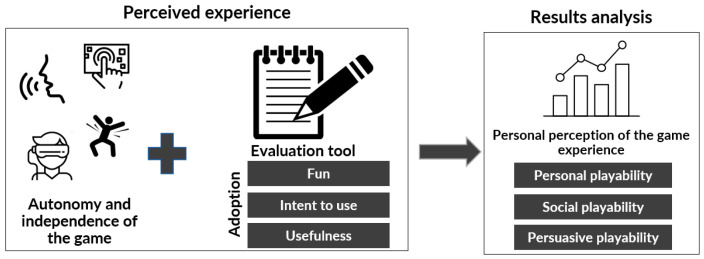
Perceived experience phase [24].

Finally, the fourth phase, called “Perceived experience”, is executed when the older adult has reached a basic level of mastery in their interaction with the game experience. The player can explore and interact with the game according to their tastes and interests at their own pace. To achieve this, a level of difficulty that the older adult finds enjoyable is set and different types of challenges can be offered, such as meeting objectives, overcoming levels, or trying to beat personal records in terms of points or times (see Figure 13).

### 3.1. Characterization

**Figure 14:**
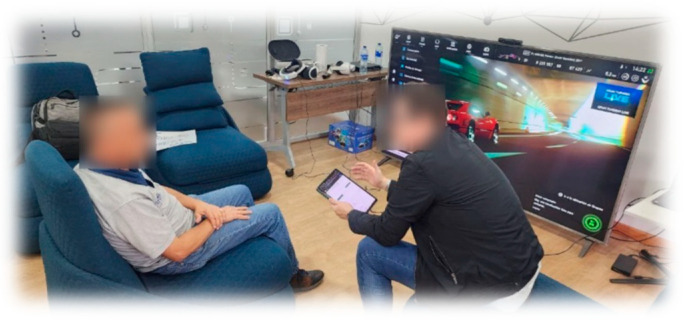
Participant characterization.

As established in the evaluation process [24], a characterization process was carried out with each of the 15 participants (because one participant was excluded from the process, as previously explained) to obtain an idea of their behavior and personal tastes in the field of games in general (see Figure 14). This characterization process, as well as all the questionnaires of each of the stages of the evaluation process, was carried out in the form of an interview, indicating to the older adult that each question should be answered on a fivee-point Likert scale, 1 being a little or not at all and 5 a lot or enough. The interview allowed us to generate a process of empathy with the participants, who commented on their personal perceptions, these being mostly neutral comments regarding the digital games.

**Figure 15:**
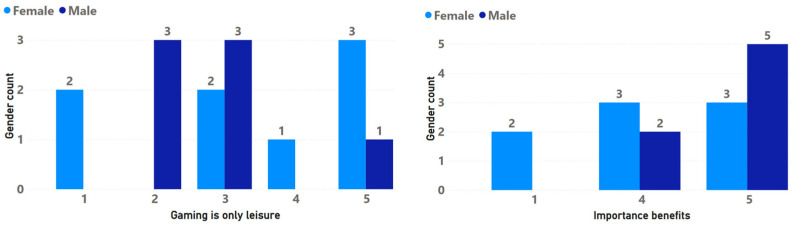
Gender analysis by leisure and benefits.

The characterization made through the interview showed that, in general, women are less familiar with digital games than men, in addition to giving less importance to this type of experience. In the family context for both genders, it was identified that digital games are important. It was possible to identify that, in general, both men and women show a greater like of social games, as well as those that focus on problem solving. Finally, opinions were mixed regarding whether digital games are simply a means of leisure and entertainment. However, when asked about the importance of a game experience providing them with benefits to justify their dedication, most responses were positive. There were few ratings that disagreed with this idea (see Figure 15).

Although the evaluation instrument with all 57 questions that are used in the “player experience evaluation” stage can be found on the PL/PX web platform, some of the questions asked in the characterization phase will be shown. Below, 5 of the 14 questions that make up this phase are shown.

- Do you feel familiar with digital games?
- In your home environment are digital games an important factor?
- Do you feel that games are just a way to pass the time?
- Do you consider that the most important thing in a game is what it can offer you in terms of knowledge, wellbeing or positive emotions?
- Do you consider that the most important thing in a game is to win?

### 3.2. Technology Training

After the characterization of the participants, technological training on PlayStation VR and Oculus Quest 2 goggles was conducted. Initially, each older adult participant was requested to use the Oculus to experience a virtual tour through various serene and relaxing places, such as beaches, snowy landscapes, and simulated forests. An important requirement was that the audio had no narration, but only relaxing music and the characteristic sounds of each place in the tour. To achieve this, a virtual tour experience available for free on YouTube was used [81]. With this, an initial technological familiarity was generated with a calm and relaxed experience that made the older adults feel comfortable and did not include any difficulty in the interaction with the technological devices. Only 1 of the 15 participants was unable to complete the virtual tour due to internet connection problems during the session, which made it necessary to omit this stage with the older adult.

In the process of selecting the game to be evaluated, two games were highlighted in the process without being the winners: Gran Turismo Sport and Danger Ball, both from PlayStation VR. These games, although they were not the focus of evaluation, were used in the process of attaining technological familiarity for the older adults in order for them to not only interact with virtual tours but also to have a first experience with quiet and simple game experiences.

**Figure 16:**
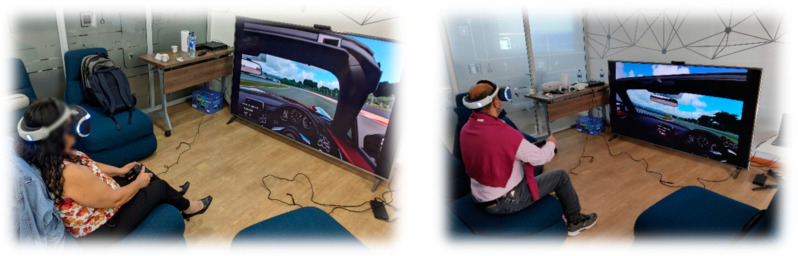
Older adults with the Gran Turismo Sport VR game experience.

Initially, the older adult was asked to play the game Gran Turismo Sport and to perform a route on the chosen track and vehicle, offering permanent accompaniment to the player while they were familiarizing and adapting to the control of the vehicle, such as acceleration, braking, and steering wheel control (see Figure 16).

This game allows for adjustments that enable an older adult to obtain a calm and relaxed gaming experience. For example, for the game test conducted with the older adults, it was determined that, instead of using the traditional commands to accelerate, brake, and turn the vehicle through the R2, L2, and joystick commands of the PlayStation 4 (PS4) DualShock controller, it would be decided to change the vehicle control to happen through the accelerometer and gyroscope to simulate the steering wheel of the car, but keeping the traditional accelerate and brake commands because these are of the trigger type and perfectly simulate the pedals of a vehicle according to the force exerted.

Another adjustment to the game experience was the vehicle that would be used, being the Mazda Roadster S 15, because it is a slow and convertible vehicle, allowing the older adult to fully see the landscapes, the asphalt, the mountains, and any other characteristic element of the landscape where driving would take place. Finally, the circuit chosen for the older adults to drive the vehicle is the one called “Sardegna”, in Italy, due to its beautiful scenery and ease of driving.

Only one participant indicated feeling dizzy during the familiarization process while driving the vehicle, being the participant who could not complete the virtual tour due to the failure of the internet connection. This highlights the importance of carrying out the complete process to generate a gradual adaptation of the participant and obtain the best possible results. Two of the participants showed a lot of enthusiasm in this first stage, so they generated an additional run, competing with themselves on the track and trying to beat their previous records.

**Figure 17:**
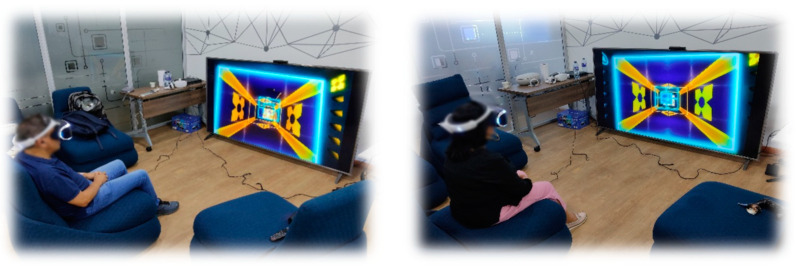
Older adults with the game experience PS Worlds: Danger Ball.

At the end of the interaction with this first game experience, the older adult was invited to interact with the second game: Danger Ball (see Figure 17). This game experience offers a quicker pace of play than the racing game, requiring more concentration and faster response times, while the first game experience required fine movements. For the execution of this game experience, some adjustments were made such as setting the game to the easy level. In addition, the game offers different opponents, each with a unique play style, and it was necessary to challenge them all and determine which ones were easy to beat. This resulted in the opponent’s named “Colossus” and “Machine” being the easiest to defeat. In this game, no participant reported feeling dizzy or uncomfortable, evidencing a greater adaptation to the VR experience.

At the end of this game experience, the questions corresponding to the evaluation stage were answered. As previously mentioned, these questions were addressed through interviews, allowing valuable information to be obtained about the interaction with the technological devices. In relation to this aspect, divided opinions were found about the benefits that can be obtained through virtual reality. Regarding the comfort of the VR devices, some participants commented that the Oculus was heavy and uncomfortable compared to PS VR.

There were also some problems with image clarity, due mainly to the poor vision of some older adults, fogging of the glasses during the game, or a lack of clarity in the video due to insufficient bandwidth during the virtual reality tour through different locations around the world. It is important to mention that these cases were uncommon. Regarding comfort during the game, there were two relevant comments. One person complained that the chair was uncomfortable, while one person mentioned that the chair was too low, which affected the proper view of the vehicle above the steering wheel. For the different comments from the participants see Section S1 of Supplementary Materials. Next, 5 of the 12 questions that were asked to the older adults in this technological training phase are presented below.

- Do you consider the technology that should be used to interact with the game experience to be easy to use?
- Do you find the posture you need to take to interact with technology comfortable?
- Do you consider the technological devices you used to be safe?
- Do you think you would interact with this technology again in the future?
- Do you consider that the use of this technology can improve your physical, cognitive health or can you obtain wellbeing through it?

### 3.3. Game Experience Training

At the end of the training with the VR devices and after identifying a basic proficiency of the participants in their interaction with these in different environments, the training on the game experience “Beat Saber” was carried out. The rules and the interaction with the controllers to control the lightsabers were explained to the older adult, all in a controlled environment.

The Beat Saber game is commercially characterized as a frenetic game that requires high physical wear and tear, but it allows for some elements to be configured to make the experience something calm and adjusted to the older adult population. First, the need to hit the boxes from a specific direction was omitted, being configured to simply hit them with the lightsaber no matter how it was achieved. Second, the speed of the game was reduced by 30% to make it slower and not require frenetic actions from the player. In addition, the punishment or loss of the game when mistakes occurred was omitted. Finally, the game was set to the easy level. To generate a symbolic and cultural familiarity with the older adults, the song “Eye of The Tiger” from “Survivor” was chosen due to its high media impact worldwide in the 80s.

**Figure 18:**
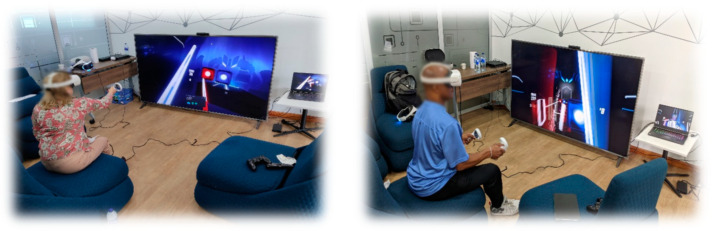
Game experience training process.

The goal of this game training was to achieve rapid familiarity with the game-based system so that, if the difficulty of the game were increased, it would produce a more immersive and enjoyable experience for the older adult (see Figure 18). No participant had problems with the training of the game, with no dizziness or sensation of discomfort being noted. This game was the one that generated the most physical activity in the participants, with it being necessary in almost all cases to drink water and wipe off perspiration.

At the end of the rules training process and after the game session, the questions corresponding to this stage of the evaluation process were answered. As in previous cases, they were conducted in the form of an interview, obtaining valuable information regarding the interaction with the game “Beat Saber”. The participants’ comments were varied regarding different aspects, for example, some did not see fundamental elements such as scores and information on the screen, others did see them, and others, although they saw them, did not understand it.

Regarding the rewards, one participant felt the reward was in the feeling of destruction when hitting the colored cubes, others felt the sounds, and others the score, but others did not feel any kind of reward. Regarding the speed of the game, divided opinions were also found: some commented that it was too fast and others that the game was too slow, but only one indicated in this question that the song was too long. To see the different comments of the participants, see Section S2 of Supplementary Materials. Next, 5 of the 15 questions that were asked to the older adults in this training phase of the game experience are presented below.

- Do you consider the rules of the game to be adequate and entertaining?
- Do you feel that the game offers interesting rewards or prizes for accomplishing objectives?
- Do you consider the game easy to learn and of adequate difficulty?
- Did you find the game story to be coherent and make sense?
- Do you feel that the game provides you with the information you need to interact with the game?

### 3.4. Perceived Experience Evaluation

**Figure 19:**
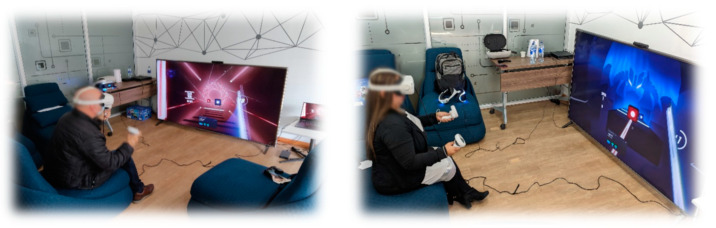
Interaction at the stage of the perceived experience evaluation.

Once the game training process has been completed, it is essential to ensure that the older adult has a clear understanding of the objective of the game, its rules, and how to interact with the necessary technological devices. Only when this understanding has been confirmed can the final stage of the process begin. This focuses on a second interaction with the game in question, but with the adjustment some parameters that make the game more challenging without overwhelming the older adult, generating an adequate flow state and increasing the immersion and fun of the participants (see Figure 19).

In this case, the 30% reduction in the speed of the game was removed, and according to the level of the skills demonstrated by the older adult in the training stage, the difficulty of the game was increased to a normal level. Also, they were asked if they wanted a different song from the one initially selected that offered a more dynamic musical rhythm, which was “Believer” by “Imagine Dragons”. This increase in difficulty and change of song was performed with five participants who quickly mastered the experience, but the 30% reduction in speed was removed for all of them.

At the end of the process of playing the game “Beat Saber” again, with the indicated adjustments, the questions corresponding to this stage of the evaluation process were answered. As in the previous cases, they were conducted in the form of an interview, obtaining valuable information about the experience offered by the game. At the end of the game experience, homogeneous responses were obtained in terms of positive experiences, time dedicated, health improvement, and well-being.

To see the different comments from the participants, see Section S3 of Supplementary Materials. It is remarkable that one of the participants was highly competitive, being insistent on being able to understand during the whole evaluation process how the victory was obtained and how they compared to the other participants. Next, 5 of the 16 questions that were asked to older adults in this phase of the evaluation of the perceived experience are presented below.

- Do you feel that the game experience allowed you to feel positive experiences away from stress, frustration, anxiety, confusion or psychological burden?
- Do you feel that the theme of the game increased your level of motivation to perform the activities through the proposed challenges?
- Did you find the experience you had interesting and useful?
- Was the use of the technological devices during the game experience enjoyable and intuitive?
- Do you consider that you had fun during the game experience?

## 4. Results and Discussion

As noted previously, 15 of the 16 participants were able to complete the entire player experience evaluation process. The evaluation process lasted, per participant, approximately 45 min, including the interviews and the interactions with the VR devices in the different games. The player experience metrics and indicators results are as follows.

### 4.1. Acceptance, Adoption and Training Results

**Figure 20:**
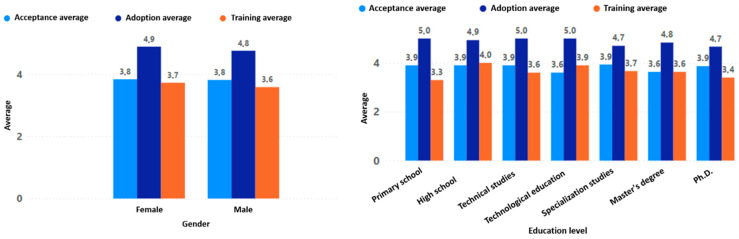
Acceptance, adoption, and training by gender and education level.

**Figure 21:**
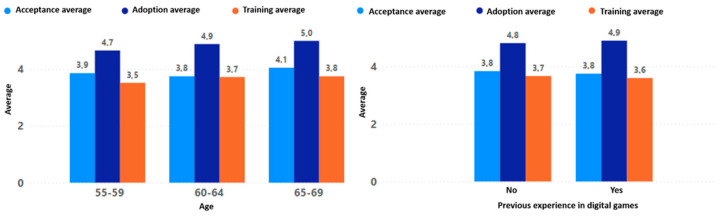
Acceptance, adoption, and training by age and previous experience.

**Figure 22:**
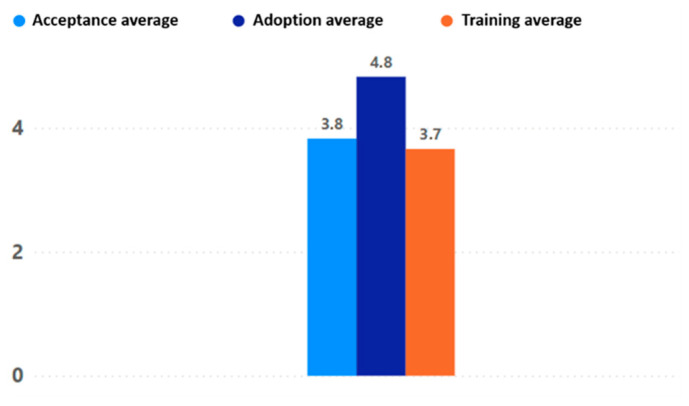
Acceptance, adoption, and training of all participants.

The results obtained for acceptance, adoption, and training throughout the evaluation process were very similar regardless of gender, education level, age, or previous experience with digital games (see Figure 20 and Figure 21). These homogeneous results are consistent with the general behavior of the entire population without segmentation by any type of filter (see Figure 22).

This evidences that the game “Beat Saber”, together with the VR devices used in the game experience, has a high degree of adoption, indicating that the participants are likely to adopt the technology and the game experience in their daily lives for the promotion of active aging. Although it has a high degree of adoption, the level of acceptance is not very high, indicating that, although the older adult accepts the dynamics and mechanics of the game experience evaluated by making use of the technology that allowed their interaction with the game, these could be improved.

Likewise, the result obtained from the training indicates that, although the older adults could interact correctly with the game experience by making proper use of the technological peripherals, they had a high margin for improvement. The specific elements that need to be improved in the game experience to obtain a better acceptance and training will be detailed in the Section 4.3.

### 4.2. Detail Adoption Results: Fun, Intent to Use and Usefulness

The results for fun, intention to use, and usefulness reflect in detail the adoption behavior of the game experience. Overall, the fun scores were very high, indicating that all participating older adults had fun with the game “Beat Saber”. The high use intent obtained indicates that older adults are really interested in continuing to use the game experience in their daily lives. Finally, the usefulness, which indicates how useful the older adult considers the game experience, was also positive, although with some differences that will be explained below.

**Figure 23:**
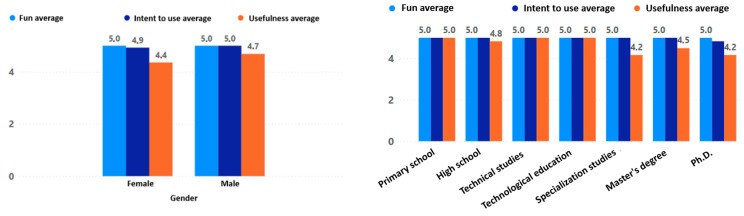
Fun, intention to use, and usefulness by gender and education level.

**Figure 24:**
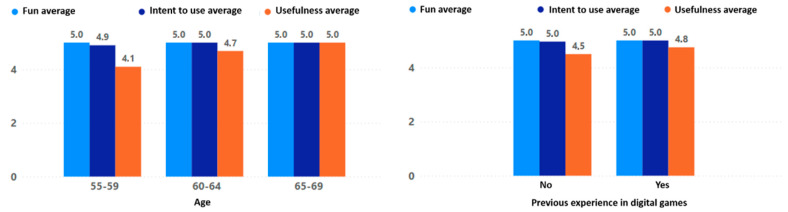
Fun, intention to use, and usefulness by age and previous experience.

The level of feeling of usefulness of the game experience was higher in women than in men; moreover, the feeling of usefulness was lower as the participant’s level of education increased (see Figure 23). Regarding age there was also an interesting behavior, with the perceived usefulness of the game experience increasing directly with the participant’s age. Finally, there was a minor increase in the perceived usefulness of the game experience when there was prior experience with digital games, but it was not very significant (see Figure 24).

To analyze the change in perception and opinion of the game experience in VR, a comparison was made between the initial and final appraisals of the process. For this purpose, the general averages of three elements that are characteristic of the beginning of the experiment were calculated: familiarity with digital games, the importance they represent in the daily life of older adults, and the time dedicated to this type of game experience in their daily routine. The values obtained for these appraisals were low, with an average of 2.47 for familiarity with digital games, 1.80 for the importance that these games represent in their lives, and 1.40 for the time dedicated to this type of game experience in their daily routine.

**Figure 25:**
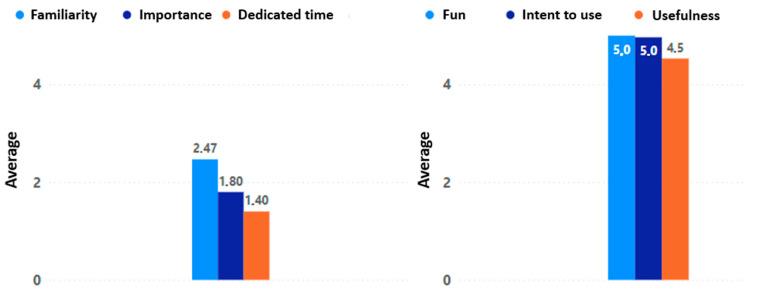
Initial perception (familiarity, importance, and dedicated time) versus final perception (fun, intent to use, and usefulness).

When comparing the final adoption results, specifically in terms of the fun, intention to use, and usefulness of this type of experience, a notable increase and positive change in perception can be observed. Both fun and intention to use were rated exceptionally high, with an average of 5.0, while usefulness obtained an average of 4.5. These results clearly show that the applied process generated a positive impact on the participants and achieved a significant change in their perception towards this type of technology (see Figure 25).

### 4.3. Playability Results from the Perspective of Older Adults

During the evaluation process conducted with older adults, the questions asked are a concise assessment tool that provides a comprehensive analysis of the game experience across the nine facets of playability [24]. The objective of this evaluation is to determine not only the PX, but also to obtain a perspective on the quality of the product from the perspective of the player. The results obtained were accurate and reflected the qualitative comments of the participants. The social facet and attribute received the lowest rating, mainly because the game “Beat Saber” only allows online multiplayer gameplay. The average of the results obtained for each facet and attribute can be found in Table 1, where the results of satisfaction (Sa), learning (Le), effectiveness (Ef), immersion (Im), motivation (Mo), emotion (Em), and socialization (Soc) are shown.

**Figure 26:**
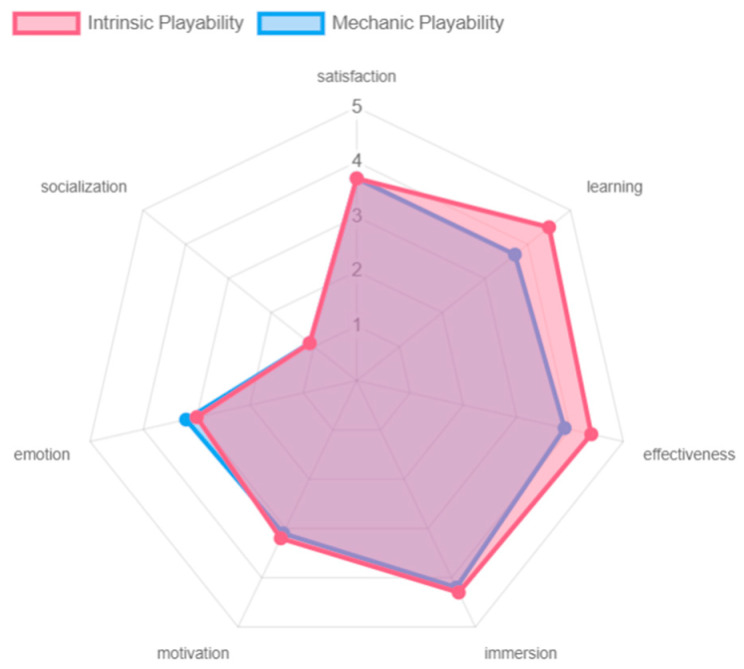
Playability intrinsic and mechanic results by older adults.

The intrinsic and mechanic playability of the game experience obtained similar results with respect to the different playability attributes (see Figure 26). From both perspectives, the game experience offers satisfaction and immersion to the participants. At the level of learning and effectiveness, the intrinsic playability received high ratings. Although the mechanic playability also received favorable scores, these were slightly lower due to a lack of clarity for one of the participants as to the objective of the game, which was to hit the cubes with the colored sabers. This affected the overall outcome of the evaluation. At the level of emotion and motivation, although the game was appreciated, this factor could be improved. The game, being based on music and rhythm, generates positive feelings in the player, but more elements could be added to intensify these feelings in the participants. In terms of socialization, the results were negative, since no clear mechanics are offered to play with other participants, as this requires an online connection and greater skills and mastery of the game experience by older adults.

**Figure 27:**
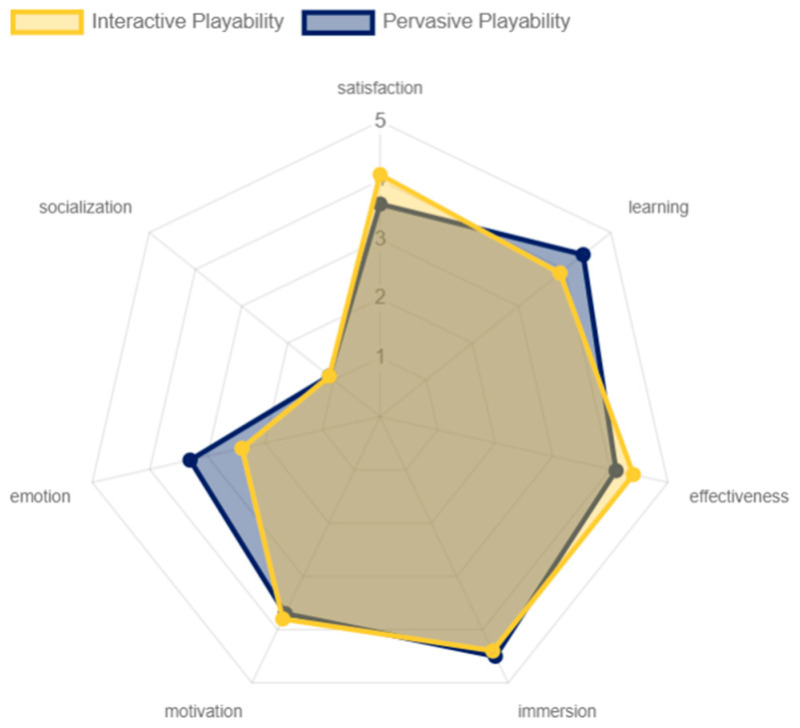
Playability pervasive and interactive results by older adults.

The results for the pervasive and interactive playability were, overall, similar. Both modalities obtained high levels of satisfaction, motivation, immersion, learning, and effectiveness. However, it is important to note that a significant difference was found in the emotional aspect between both experiences. The interactive playability showed a slightly lower score compared to the pervasive playability. This was due to the limited variety of game objectives offered to the older adults, as the experience was focused on hitting the cubes with the lightsaber. In contrast, the pervasive playability provided a richer and more diverse experience. In terms of the social dimension, both approaches were rated negatively, as mentioned previously (ver Figure 27). 

**Figure 28:**
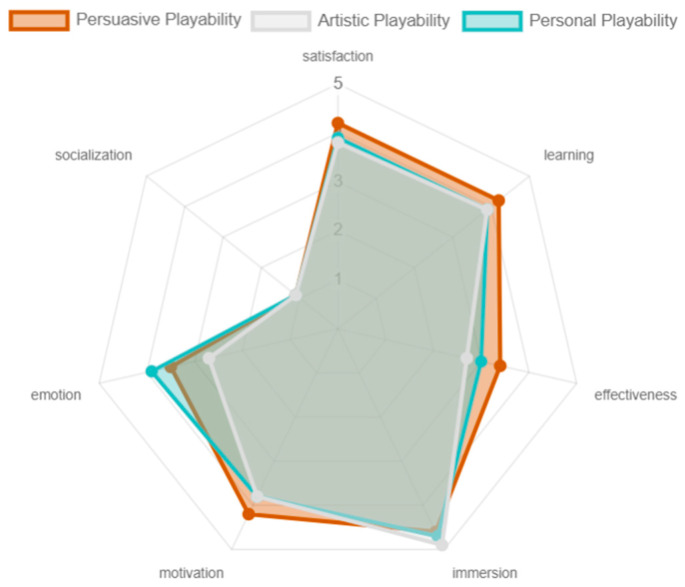
Playability artistic, personal, and persuasive results by older adults.

The artistic, personal, and persuasive playability obtained similar results, showing differences only in emotion. The satisfaction, learning, immersion, and motivation were positively evaluated. Regarding emotion, the only one that was not positively valued was the artistic playability, this because, although the game offers pleasant colors and transitions, there is no strong symbolic and cultural familiarity that strengthens the emotion even more which is generated by hitting the colored cubes. This, too, is reflected in the effectiveness of the game experience with respect to comparative playability (see Figure 28).

**Figure 29:**
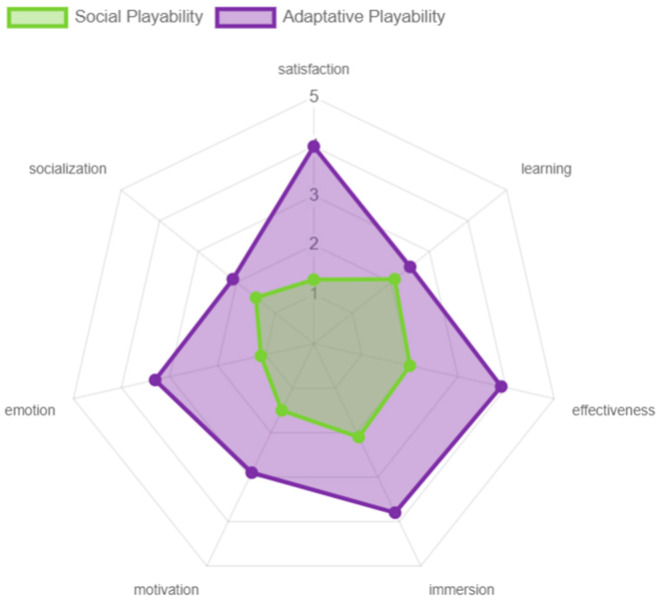
Playability adaptative and social results by older adults.

Regarding the adaptive playability, this had a different behavior from all the others, with the satisfaction, effectiveness, immersion, and excitement that virtual reality, through the game “Beat Saber”, offered to the participants being positively valued. At the learning level, this was not so positively valued, because the game does not offer an introductory tutorial or intuitive guides for older adults, and as they expressed during the experience, they feel they achieved a smooth interaction due to the constant accompaniment and guidance, not because the game instructed them in the process. This is also reflected in motivation, since the game itself did not motivate them to play or socialize with other people. Finally, the social playability was rated poorly in all aspects, due to the reasons discussed above (see Figure 29).

Based on the results obtained, the PL/PX web platform provided a series of 4 recommendations and identified 12 critical issues in the game experience. These recommendations not only seek to improve the playability of the “Beat Saber” game experience, but also to increase acceptance, training, and adoption. Improving adoption has a direct impact on the fun, acceptance, and intent to use the game experience. A list of these recommendations and critical issues can be found in Section S4 of Supplementary Materials.

Considering the high level of adoption obtained in the evaluation of the selected game experience, it is evident that its characteristics allowed it to be sufficiently adaptive to be enjoyed by the older adult population. In addition, this experience promotes physical activity and cognitive training through coordination. Therefore, it is possible to conclude that the evaluation process was successful.

**Figure 30:**
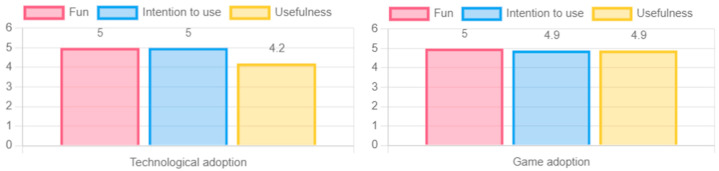
Results in terms of fun, intention of use, and usefulness of the game and technology.

This adoption is supported by the different results obtained in terms of fun, usefulness, and intention to use not only the game experience, but also the VR devices used. As can be seen in Figure 30, the fun rating by all participants was high, both in the game experience “Beat Saber” and in other scenarios offered by virtual reality. Likewise, the usefulness was positively valued, being reinforced when the older adults interacted with the game experience, all this reflecting a positive intention of use for the promotion of active aging.

Finally, an accurate and objective evaluation was carried out, which made it possible to identify critical elements that negatively affected the game experience. In addition, recommendations were formulated with the aim of significantly improving the fun and, thus, providing a GBS more adjusted to the tastes and particularities of the elderly population. All this progress was possible due to the implementation of a meticulous evaluation process, which included detailed steps for technological training and adjustment of the game rules, ensuring that these were well received by the players.

### 4.4. Results Comparison: Experts vs. Older Adults

The established evaluation process offers two perspectives on playability analysis: experts and players [24]. Although both provide an analysis of playability, the expert evaluation tool is much more complete and more extensive. In its complete version, it has a total of 246 evaluation items, while the one made for the players consists of only 43 items, without considering the characterization, since it does not focus on evaluating the game experience. Despite this difference, the results obtained from both points of view are similar, although with slight differences. These discrepancies are justified and explained by the way in which the technological training and game process was carried out in a controlled environment conducive to the enjoyment of the older adult population. The following is a comparison of the results obtained by the set of experts in relation to the results obtained by all participants for the nine facets of playability.

**Figure 31:**
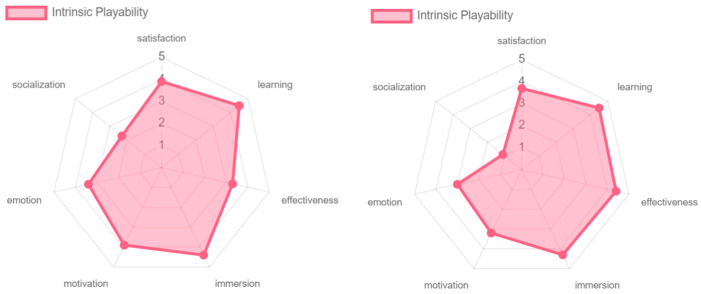
Intrinsic playability: experts vs. older adults.

**Figure 32:**
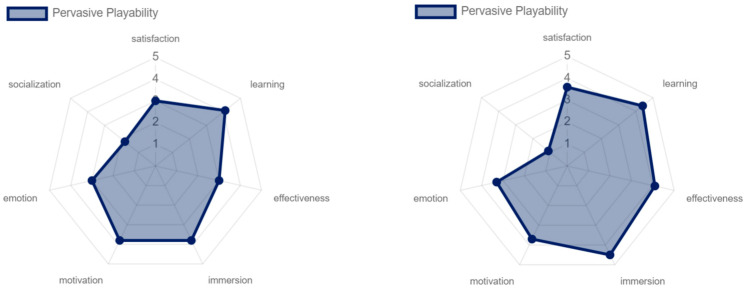
Pervasive playability: experts vs. older adults.

Regarding intrinsic and pervasive playability, the older adults evaluated the effectiveness of the game very positively due to the constant guidance and support they received. This included the necessary technological training as well as the rules of the game itself (see Figure 31 and Figure 32).

**Figure 33:**
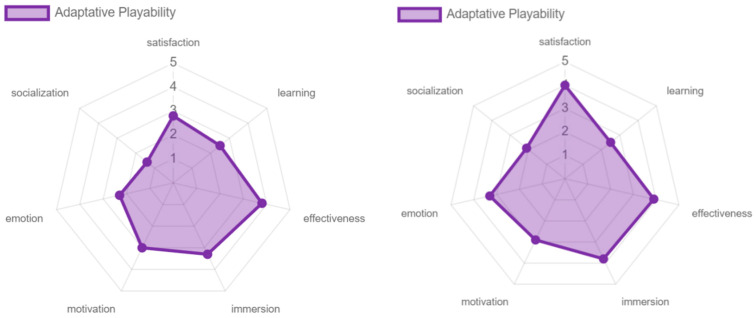
Adaptative playability: experts vs. older adults.

In the pervasive and adaptative playability, player satisfaction was rated more positively, since having an environment that is controlled in terms of the music, the level of difficulty, and various elements aimed at obtaining the greatest possible game flow positively affects player satisfaction (see Figure 32 and Figure 33).

**Figure 34:**
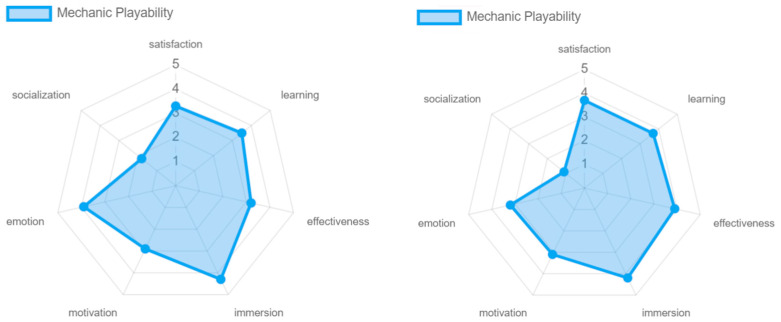
Mechanic playability: experts vs. older adults.

**Figure 35:**
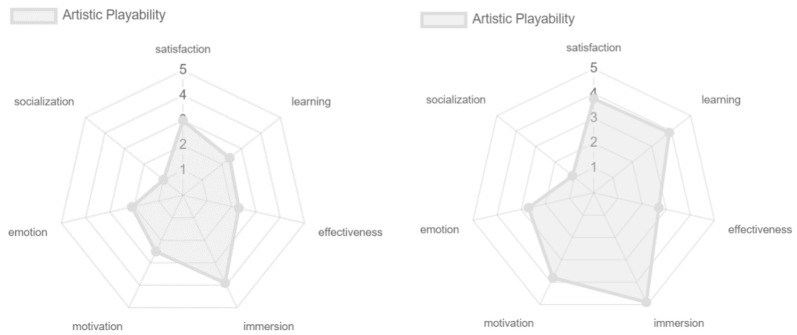
Artistic playability: experts vs. older adults.

**Figure 36:**
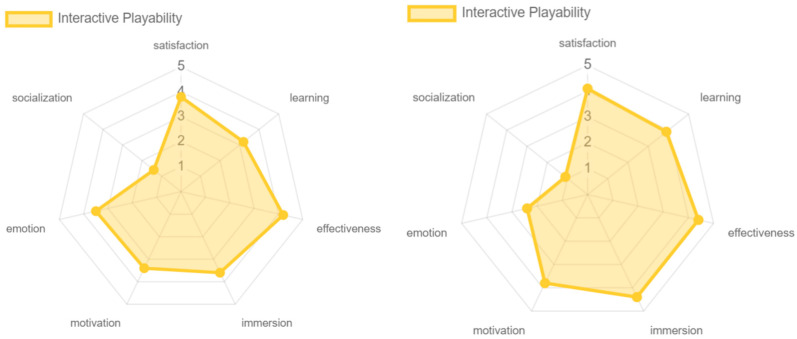
Interactive playability: experts vs. older adults.

**Figure 37:**
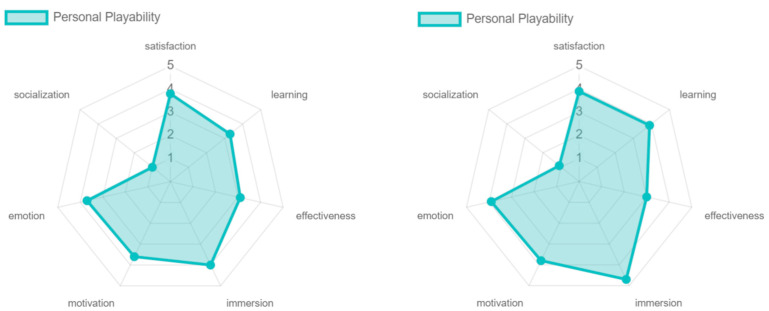
Personal playability: experts vs. older adults.

Regarding the social aspect, although, in general, it was very poorly rated, some differences were found, as in the case of intrinsic, pervasive, mechanic, and persuasive playability, in which the experts rated it a little more positively than the older adult population, because the experts knew about the existence of the online game, while the older adults did not (see Figure 31, Figure 32 and Figure 34). With respect to emotion, this was better valued in terms of the adaptive and artistic playability by older adults, but it should be noted that, as in previous cases, offering an ideal environment conducive to the enjoyment of the experience by the older adult population may have slightly affected the results (see Figure 33 and Figure 35). Regarding learning, this was valued more positively by the older adults in terms of the artistic, interactive, and personal playability, since, although the game does not make use of guides and tutorials, the constant help during the process favored the participants’ learning of the game experience. This indirectly affected the artistic playability with respect to other attributes such as satisfaction, emotion, and immersion (see Figure 35, Figure 36 and Figure 37).

**Figure 38:**
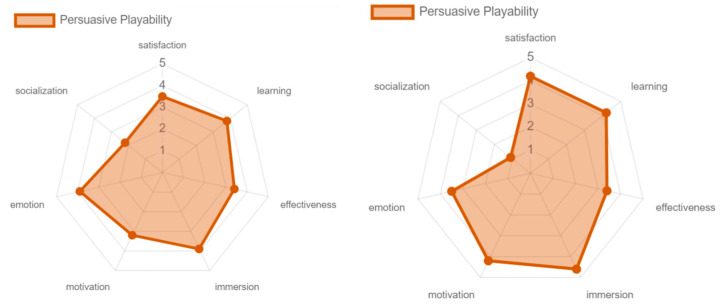
Persuasive playability: experts vs. older adults.

**Figure 39:**
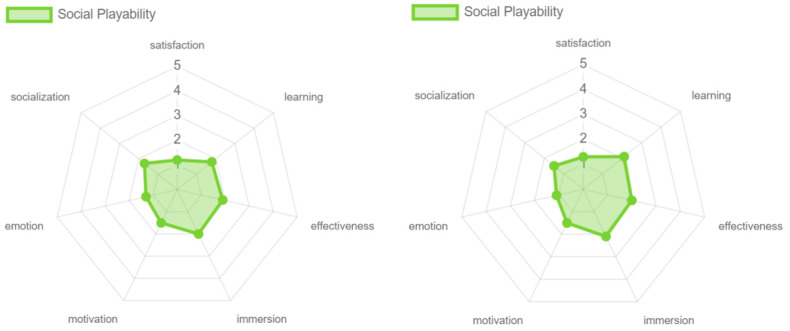
Social playability: experts vs. older adults.

Regarding motivation, the older adults valued persuasive playability more highly, mainly due to two key factors in the controlled environment. First, music that they could associate with their lifestyle was used, which helped to generate an emotional connection to the game. Second, during the process, the benefits that the game could bring to active aging were emphasized, without the game experience focusing on highlighting these aspects, affecting the results obtained (see Figure 38). Finally, very similar results were obtained in all the attributes of the social playability facet, without major changes being found, coinciding with the low results obtained (see Figure 39).

## 5. Conclusions

Defining a process for evaluating game experiences and fun for older adults has been a significant challenge. The target population is a heterogeneous group with different tastes and particularities, which makes it difficult to create a process that is suitable for everyone. However, it was possible to identify a series of particularities common to this generational population, which provided the basis for the formulation and definition of a complete evaluation process. This process includes aspects from the design of a game experience to evaluation by the end users. Due to its complexity, it has been divided into phases to obtain the best possible results and to detail it in the best possible way. To date, the first and second phases of the evaluation process have been completed.

To validate the third stage of the proposed process, the game chosen to be evaluated was the game called “Beat Saber”. This game was evaluated by a total of 15 older adult participants, applying the established evaluation process and obtaining positive results in terms of fun, intention to use, and usefulness. The considerable number of possible problems identified shows the potential of the heuristics and checklists defined. The high similarity between the results obtained by the experts and by the older adults regarding the evaluation of fun and the quality of the game as a product concludes the completeness and effectiveness of the entire evaluation process as defined.

The interaction of the older adults with the chosen game was an enriching experience. It was possible to apply a complete validation, including the profiling of the participants, a training process of the technology to be used, and explaining the rules of the game; finally, when they had a basic proficiency in it, they could play according to their personal preferences. This evaluation process could be carried out in a calm and controlled environment, generating confidence in the players. This was key in the process, since it allowed us to obtain objective results from the participants.

In addition, training in the use of technology played a key role in improving older adults’ acceptance of technology. This training not only increased the perceived usefulness and perceived ease of use, but also increased the perceived enjoyment, all factors that influence attitudes toward technology use. A positive attitude toward use is a strong predictor of intention to use, suggesting that providing older adults with an appropriate learning environment may be critical to technology adoption.

The results also highlight the influence of external factors such as the subjective norm and facilitating conditions. Perceived social pressure and support from family and friends significantly influence the willingness of older adults to adopt new technologies. In addition, facilitating conditions, such as technological infrastructure, available technical support, and continuous training, are critical to the perceived ease of use.

On the other hand, individual user characteristics, such as previous experience with technology, technology anxiety, and computer autonomy, also significantly affect technology acceptance. These results underscore the importance of considering both internal and external factors when assessing technology acceptance in older adults. A comprehensive strategy that strengthens these types of factors may encourage the greater adoption of new technologies in this demographic group, facilitating their integration and use in daily life. It would have been desirable to have a larger population group in order to offer more solid results, but the results obtained herein agree with the results and appreciations obtained previously by a group of experts.

As future work, our expectations are to evaluate the fun and perceived experience in GBS using different pervasive technologies, such as geo-references, augmented reality, virtual assistants, or tabletops. Also, it is expected to evaluate the impact of other types of game experiences for the promotion of active aging that are not pervasive. In addition, it is expected for our framework to also be applied for other more specific purposes such as health improvement or application in therapies for the treatment of diseases. Finally, based on the results of playability and pervasiveness expansion achieved in the methodological evaluation process, it is expected to make a general-purpose proposal without focusing on a specific generational group, thus covering a broad population group.

## Figures and Tables

**Table 1:** Playability analysis results by older adults.

Playability/Attributes	Sa	Le	Ef	Im	Mo	Em	Soc	Average
Intrinsic	3.7	4.5	4.4	4.3	3.2	3.0	1.1	3.4
Mechanic	3.7	3.7	3.9	4.2	3.1	3.2	1.1	3.2
Interactive	4.1	3.9	4.4	4.4	3.8	2.4	1.1	3.4
Artistic	3.8	3.9	2.7	4.9	3.8	2.7	1.1	3.2
Personal	3.9	3.9	3.0	4.7	3.8	3.9	1.1	3.4
Social	1.3	2.1	2.0	2.1	1.5	1.1	1.5	1.6
Adaptative	4.0	2.5	3.9	3.8	2.9	3.3	2.1	3.2
Persuasive	4.2	4.2	3.4	4.6	4.2	3.5	1.1	3.6
Pervasive	3.6	4.4	4.1	4.5	3.7	3.3	1.1	3.5
Average	3.5	3.6	3.5	4.1	3.3	2.9	1.2	

## Data Availability

Data are contained within the article.

## Supplementary Materials

A complete list of the qualitative results of the evaluation is available at: https://1drv.ms/b/s!AkaBCG8z281S5mh8O1-YdER2BQN0?e=pvobv1 (accessed on 10 October 2023).

## References

[1] Carstensen L.L. (1995). Evidence for a Life-Span Theory of Socioemotional Selectivity. Curr. Dir. Psychol. Sci..

[2] Possler D., Klimmt C., Schlütz D., Walkenbach J. (2017). A Mature Kind of Fun? Exploring Silver Gamers’ Motivation to Play Casual Games—Results from a Large-Scale Online Survey. Lecture Notes in Computer Science (Including Subseries Lecture Notes in Artificial Intelligence and Lecture Notes in Bioinformatics).

[3] D’Haeseleer I., Schoofs J., Schutters K., Schreurs D., Abeele V. (2022). Influence of Motivational Design Techniques on Use and Acceptance of Self-Management Health Systems in Older Adults. EAI Endorsed Trans. Pervasive Health Technol..

[4] Salazar-Cardona J., Gutiérrez Vela F.L., Arango-Lopez J., Moreira F. (2023). Older adults’ motivations in game based systems: Heuristic definition and its connection with fun. Comput. Hum. Behav. Rep..

[5] Sansone C., Harackiewicz J. (2000). Intrinsic and Extrinsic Motivation: The Search for Optimal Motivation and Performance.

[6] Smeddinck J.D., Herrlich M., Malaka R. Exergames for Physiotherapy and Rehabilitation: A Medium-Term Situated Study of Motivational Aspects and Impact on Functional Reach. Proceedings of the 33rd Annual ACM Conference on Human Factors in Computing Systems. Association for Computing Machinery.

[7] Seaborn K., Pennefather P., Fels D.I. (2020). Eudaimonia and Hedonia in the Design and Evaluation of a Cooperative Game for Psychosocial Well-Being. Hum. Comput. Interact..

[8] Kappen D.L., Nacke L.E., Gerling K.M., Tsotsos L.E. Design strategies for gamified physical activity applications for older adults. Proceedings of the Annual Hawaii International Conference on System Sciences.

[9] Marcos-Pardo P.J., Martínez-Rodríguez A., Gil-Arias A. (2018). Impact of a motivational resistance-training programme on adherence and body composition in the elderly OPEN. Sci. Rep..

[10] De Vries A.W., Van Dieën J.H., Van Den Abeele V., Verschueren S.M.P. (2018). Understanding Motivations and Player Experiences of Older Adults in Virtual Reality Training. Games Health J..

[11] Altmeyer M., Lessel P., Krüger A. Investigating gamification for seniors aged 75+. Proceedings of the 2018 Designing Interactive Systems Conference.

[12] Razak F.H.A., Azhar N.H.C., Adnan W.A.W., Nasruddin Z.A. (2017). Exploring malay older user motivation to play mobile games. Advances in Visual Informatics, Proceedings of the 5th International Visual Informatics Conference, IVIC 2017, Bangi, Malaysia, 28–30 November 2017.

[13] Chesham A., Wyss P., Müri R.M., Mosimann U.P., Nef T. (2017). What older people like to play: Genre preferences and acceptance of casual games. JMIR Serious Games.

[14] De Carvalho R.N.S., Ishitani L. (2012). Motivational Factors for Mobile Serious Games for Elderly Users. XI Simpósio Bras. Jogos E Entretenimento Digit.-SBGames.

[15] Nap H.H., de Kort Y.A.W., Ijsselsteijn W.A. (2009). Senior gamers: Preferences, motivations and needs. Gerontechnology.

[16] Yang H.L., Lin S.L. (2019). The reasons why elderly mobile users adopt ubiquitous mobile social service. Comput. Hum. Behav..

[17] Ryan R.M., Deci E.L. (2000). Self-determination theory and the facilitation of intrinsic motivation, social development, and well-being. Am. Psychol..

[18] Subramanian S., Dahl Y., Skjæret Maroni N., Vereijken B., Svanæs D. (2020). Assessing Motivational Differences between Young and Older Adults When Playing an Exergame. Games Health J..

[19] Khalili-Mahani N., de Schutter B., Sawchuk K. (2020). The Relationship between the Seniors’ Appraisal of Cognitive-Training Games and Game-Related Stress Is Complex: A Mixed-Methods Study. Lecture Notes in Computer Science (Including Subseries Lecture Notes in Artificial Intelligence and Lecture Notes in Bioinformatics).

[20] Khalili-Mahani N., De Schutter B. (2019). Affective game planning for health applications: Quantitative extension of gerontoludic design based on the appraisal theory of stress and coping. JMIR Serious Games.

[21] Carstensen L.L., Hartel C.R. (2006). When I’m 64.

[22] Davis F.D. (1985). Technology Acceptance Model for Empirically Testing New End-User Information Systems: Theory and Results.

[23] Venkatesh V., Morris M.G., Davis G.B., Davis F.D. (2003). User acceptance of information technology: Toward a unified view. MIS Q..

[24] Salazar-Cardona J.A., Gutierrez F.L., Arango-López J. (2024). Proceso de Evaluación de Experiencias de Juego y Diversión en Adultos Mayores Que Involucre Pervasividad Para Fomentar el Envejecimiento Activo.

[25] Salazar-Cardona J.A., Cano S., Gutiérrez-Vela F.L., Arango J. (2023). Designing a Tangible User Interface (TUI) for the Elderly Based on Their Motivations and Game Elements. Sensors.

[26] Salazar-Cardona J., Arango-López J., Gutiérrez-Vela F.L. PL/PX Platform: Online tool for the evaluation of fun and game experiences. Proceedings of the Conference: II Congreso Español de Videojuegos.

[27] Hermawati S., Lawson G. (2015). A user-centric methodology to establish usability heuristics for specific domains. Contemp. Ergon. Hum. Factors.

[28] (2019). Ergonomics of Human-System Interaction—Part 210: Human-Centred Design for Interactive Systems.

[29] González J.L., Gutierrez F.L. (2010). Jugabilidad: Caracterización de la Experiencia del Jugador en Videojuegos.

[30] Rienzo A., Cubillos C. (2020). Playability and player experience in digital games for elderly: A systematic literature review. Sensors.

[31] Procci K., Singer A.R., Levy K.R., Bowers C. (2012). Measuring the flow experience of gamers: An evaluation of the DFS-2. Comput. Human Behav..

[32] González Sánchez J.L., Padilla Zea N., Gutiérrez F.L., Montero F. Jugabilidad como Calidad de la Experiencia del Jugador en Videojuegos. Proceedings of the Actas del X Congreso Internacional de Interaccion Persona-Ordenador, INTERACCION.

[33] Arango-López J., Collazos C.A., Gutiérrez Vela F.L., Castillo L.F. (2017). A systematic review of geolocated pervasive games: A perspective from game development methodologies, software metrics and linked open data. Lecture Notes in Computer Science (Including Subseries Lecture Notes in Artificial Intelligence and Lecture Notes in Bioinformatics).

[34] Drachen A., Nacke L. Towards a Framework of Player Experience Research. Proceedings of the Second International Workshop on Evaluating Player Experience in Games at FDG.

[35] Isbister K., Schaffer N. (2008). The Four Fun Keys. Game Usability.

[36] Salazar Cardona J., Gutiérrez Vela F.L., Lopez Arango J., Gallardo J. (2021). Game-based systems: Towards a new proposal for playability analysis. CEUR Workshop Proc..

[37] Brooke J. (2020). SUS: A “Quick and Dirty” Usability Scale. Usability Eval. Ind..

[38] De Paula G., Valentim P., Seixas F., Santana R., Muchaluat-Saade D. Sensory effects in cognitive exercises for elderly users: Stroop game. Proceedings of the 2020 IEEE 33rd International Symposium on Computer-Based Medical Systems (CBMS).

[39] Muñoz G.F., Cardenas R.A.M., Pla F. (2021). A kinect-based interactive system for home-assisted active aging. Sensors.

[40] Adcock M., Sonder F., Schättin A., Gennaro F., de Bruin E.D. (2020). A usability study of a multicomponent video game-based training for older adults. Eur. Rev. Aging Phys. Act..

[41] Adcock M., Thalmann M., Schättin A., Gennaro F., de Bruin E.D. (2019). A Pilot Study of an In-Home Multicomponent Exergame Training for Older Adults: Feasibility, Usability and Pre-Post Evaluation. Front. Aging Neurosci..

[42] Boj C., Díaz D.J., Portalés C., Casas S. (2018). Video games and outdoor physical activity for the elderly: Applications of the HybridPLAY technology. Appl. Sci..

[43] Jansen-Kosterink S., Bulthuis R., Stal S.T., van Velsen L., Pnevmatikakis A., Kyriazakos S., Pomazanskyi A., den Akker H.O. (2020). The results of an iterative evaluation process of an mhealth application for rewarding healthy behaviour among older adults. Communications in Computer and Information Science.

[44] Guimarães V., Pereira A., Oliveira E., Carvalho A., Peixoto R. Design and evaluation of an exergame for motor-cognitive training and fall prevention in older adults. Proceedings of the 4th EAI International Conference on Smart Objects and Technologies for Social Good.

[45] Silva J., Oliveira E., Moreira D., Nunes F. (2018). Design and Evaluation of a Fall Prevention Multiplayer Game for Senior Care Centres. Lecture Notes in Computer Science (Including Subseries Lecture Notes in Artificial Intelligence and Lecture Notes in Bioinformatics).

[46] Chu C.H., Biss R.K., Cooper L., Quan A.M.L., Matulis H. (2021). Exergaming platform for older adults residing in long-term care homes: User-centered design, development, and usability study. JMIR Serious Games.

[47] Pyae A. Investigating the Usability, User Experiences, and Usefulness of Digital Game-based Exercises for Elderly People: A case study of Finland. Proceedings of the 2018 Annual Symposium on Computer-Human Interaction in Play Companion Extended Abstracts.

[48] Ahmad F., Zongwei L., Ahmed Z., Muneeb S. (2020). Behavioral profiling: A generationwide study of players’ experiences during brain games play. Interact. Learn. Environ..

[49] Davis F.D. (1989). Perceived usefulness, perceived ease of use, and user acceptance of information technology. MIS Q..

[50] Borrego G., Morán A.L., Meza V., Orihuela-Espina F., Sucar L.E. (2020). Key factors that influence the UX of a dual-player game for the cognitive stimulation and motor rehabilitation of older adults. Univers. Access Inf. Soc..

[51] Yu R.W.L., Yuen W.H., Peng L., Chan A.H.S. (2020). Acceptance Level of Older Chinese People Towards Video Shooting Games. Lecture Notes in Computer Science (Including Subseries Lecture Notes in Artificial Intelligence and Lecture Notes in Bioinformatics).

[52] Venkatesh V., Thong J.Y.L., Xu X. (2012). Consumer acceptance and use of information technology: Extending the unified theory of acceptance and use of technology. MIS Q..

[53] Oppl S., Stary C. (2020). Game-playing as an effective learning resource for elderly people: Encouraging experiential adoption of touchscreen technologies. Univers. Access Inf. Soc..

[54] Shao D., Lee I.J. (2020). Acceptance and influencing factors of social virtual reality in the urban elderly. Sustainability.

[55] Merilampi S., Mulholland K., Ihanakangas V., Ojala J., Valo P., Virkki J. A Smart Chair Physiotherapy Exergame for Fall Prevention—User Experience Study. Proceedings of the 2019 IEEE 7th International Conference on Serious Games and Applications for Health.

[56] Greipl S., Moeller K., Kiili K., Ninaus M. (2020). Different performance, full experience: A learning game applied throughout adulthood. Int. J. Serious Games.

[57] Pereira F., Bermudez IBadia S., Jorge C., Da Silva Cameirao M. Impact of Game Mode on Engagement and Social Involvement in Multi-User Serious Games with Stroke Patients. Proceedings of the International Conference on Virtual Rehabilitation, ICVR 2019.

[58] Santos L.H., Okamoto K., Hiragi S., Yamamoto G., Sugiyama O., Aoyama T., Kuroda T. (2019). Pervasive game design to evaluate social interaction effects on levels of physical activity among older adults. J. Rehabil. Assist. Technol. Eng..

[59] Pyae A., Joelsson T., Saarenpää T., Luimula M., Kattimeri C., Pitkäkangas P., Granholm P., Smed J. (2017). Lessons Learned from Two Usability Studies of Digital Skiing Game with Elderly People in Finland and Japan. Int. J. Serious Games.

[60] Leite Araujo R., Da Silva Sena T., Takako Endo P. (2021). Gamification applied to an elderly monitoring system during the COVID-19 pandemic. IEEE Lat. Am. Trans..

[61] Xu W., Liang H.N., Yu K., Baghaei N. Efect of Gameplay Uncertainty, Display Type, and Age on Virtual Reality Exergames. Proceedings of the 2021 CHI Conference on Human Factors in Computing Systems.

[62] Ijsselsteijn W.A., Kort D., Poels Y.A.W. (2013). Game Experience Questionnaire.

[63] Ryan R., Rigby S., Przybylski A. (2006). The Player Experience of Need Satisfaction (PENS).

[64] McAuley E.D., Duncan T., Tammen V.V. (2013). Psychometric Properties of the Intrinsic Motivation Inventory in a Competitive Sport Setting: A Confirmatory Factor Analysis. Res. Q. Exerc. Sport.

[65] Crawford J.R., Henry J.D. (2004). The Positive and Negative Affect Schedule (PANAS): Construct validity, measurement properties and normative data in a large non-clinical sample. Br. J. Clin. Psychol..

[66] Murciano Hueso A., Martín García A.V., Torrijos Fincias P. (2022). Revisión sistemática de aceptación de la tecnología digital en personas mayores. Perspectiva de los modelos TAM Rev. Esp. Geriatr. Gerontol..

[67] Huang L. (2003). The Impact of Cultural Values on Email Acceptance: Evidence from the PRC. Ph.D. Thesis.

[68] Lee S., Kim B.G. (2009). Factors affecting the usage of intranet: A confirmatory study. Comput. Hum. Behav..

[69] Straub D., Keil M., Brenner W. (1997). Testing the technology acceptance model across cultures: A three country study. Inf. Manag..

[70] Venkatesh V., Davis F.D. (2000). A Theoretical Extension of the Technology Acceptance Model: Four Longitudinal Field Studies. Manag. Sci..

[71] (2011). Technology Acceptance in Education. https://users.ugent.be/~wduyck/articles/PynooDevolderTondeurVanBraakDuyckDuyck2011b.pdf.

[72] Yan G., Xu M. (2004). An Enhanced Technology Acceptance Model for Web-Based Learning. J. Inf. Syst. Educ..

[73] George Saadé R., Kira D. (2006). The Emotional State of Technology Acceptance. Issues Informing Sci. Inf. Technol..

[74] Nielsen J., Molich R. Heuristic evaluation of user interfaces. Proceedings of the Conference on Human Factors in Computing Systems.

[75] Salazar J.A., Arango J., Gutiérrez F.L., Moreira F. (2022). Older Adults and Games from a Perspective of Playability, Game Experience and Pervasive Environments: A Systematics Literature Review. World Conference on Information Systems and Technologies.

[76] Kitchenham B., Charters S. (2007). Guidelines for performing Systematic Literature Reviews in Software Engineering. Engineering.

[77] Quiñones D., Rusu C., Rusu V. (2018). A methodology to develop usability/user experience heuristics. Comput. Stand Interfaces.

[78] Cardona J.S., Vela F.L.G., Lopez J.A., Moreira F. (2024). Heuristics for Designing Pervasive Game Experiences in the Older Adult Population. Good Practices and New Perspectives in Information Systems and Technologies. WorldCIST 2024.

[79] Salazar Cardona J., Arango Lopez J., Gutiérrez Vela F.L., Moreira F. (2023). Considerations in the design of pervasive game-based systems for the older adult population. World Conference on Information Systems and Technologies.

[80] Salazar J., Arango J., Gutierrez F., Paderewski P. (2022). Adultos mayores y tipos de jugadores en sistemas basados en juego: Clasificación basada en sus motivaciones. Interaccion.

[81] Eco V.R. (2017). Virtual Nature 360°—5K Nature Meditation for Daydream, Oculus, Gear VR. https://www.youtube.com/watch?v=7AkbUfZjS5k&list=PLl0VAFUlZgo6Wf-sWK0IFGd3L8cM8mTut&index=6&ab_channel=ECOVR.

